# Association of Sustained Low or High Income and Income Changes With Risk of Incident Type 2 Diabetes Among Individuals Aged 30 to 64 Years

**DOI:** 10.1001/jamanetworkopen.2023.30024

**Published:** 2023-08-21

**Authors:** Jimin Clara Park, Ga Eun Nam, Jinna Yu, Ketrell L. McWhorter, Junxiu Liu, Hong Seok Lee, Seong-Su Lee, Kyungdo Han

**Affiliations:** 1 Episcopal Collegiate School, Little Rock, Arkansas; 2 Department of Family Medicine, Korea University Guro Hospital, Korea University College of Medicine, Seoul, South Korea; 3 Department of Nursing, Chung-Ang University, Seoul, South Korea; 4 Department of Epidemiology and Environmental Health, College of Public Health, University of Kentucky, Lexington; 5 Department of Population Health Science and Policy, Icahn School of Medicine at Mount Sinai, New York, New York; 6 Division of Cardiology, Banner University Medical Group, Sarver Heart Center, University of Arizona, Tucson; 7 Division of Endocrinology and Metabolism, Department of Internal Medicine, College of Medicine, The Catholic University of Korea, Seoul, South Korea; 8 Department of Statistics and Actuarial Science, Soongsil University, Seoul, South Korea

## Abstract

**Importance:**

Evidence of the association between income fluctuation and risk of type 2 diabetes (T2D) is scarce.

**Objective:**

To investigate whether sustained low or high income and income changes are associated with incidence of T2D.

**Design, Setting, and Participants:**

In this population-based cohort study, more than 7.8 million adults without T2D aged 30 to 64 years from a nationally representative sample from the Korean Health Insurance Service database were enrolled in 2012 and followed up to 2019 (median follow-up, 6.3 years [IQR, 6.1-6.6 years]).

**Exposures:**

Twenty quantiles of monthly health insurance premiums determined income levels. Income quartiles were annually analyzed from 2008 to 2012. Beneficiaries of the Medical Aid Program were regarded as those with very low income. A decrease in income was indicated as a reduction of 25% or more in income compared with income in the previous year.

**Main Outcomes and Measures:**

The primary outcome was incident T2D based on the *International Statistical Classification of Diseases and Related Health Problems, Tenth Revision* codes E11 to E14, 1 or more claims of antidiabetic medication, or a fasting glucose level of 126 mg/dL or higher. Multivariable Cox proportional hazards models were used to assess the association of low- or high-income status and income changes with incidence of T2D.

**Results:**

Of 7 821 227 participants (mean [SD] age, 46.4 [9.3] years; 54.9% men), 359 931 (4.6%) developed T2D at least 1 year after enrollment. Individuals who repeatedly experienced low and very low income for 5 years showed 22% (hazard ratio [HR], 1.22 [95% CI, 1.21-1.23]) and 57% (1.57 [95% CI, 1.53-1.62]) higher T2D risk compared with those who never experienced low and very low income, respectively. In contrast, individuals who were repeatedly in high-income quartiles showed lower T2D risk compared with those who never experienced high income (HR, 0.86 [95% CI, 0.85-0.86]). The number of income decreases was associated with elevated T2D risk (≥2 vs 0 income decreases: HR, 1.08 [95% CI, 1.06-1.11]; *P* < .001 for trend). When income quartile status was compared between 2008 and 2012, individuals who experienced an income increase had lowered T2D risk, while those who experienced an income decrease had elevated T2D risk in each income quartile group.

**Conclusions and Relevance:**

This cohort study found that individuals who experienced sustained low-income status or an income decrease had elevated T2D risk, while those who had sustained high-income status or an income increase had lowered T2D risk.

## Key Points

**Question:**

Are sustained low or high income and income changes associated with risk of type 2 diabetes (T2D)?

**Findings:**

In this population-based cohort study of more than 7.8 million adults in South Korea, individuals who had low income for 5 consecutive years had the highest T2D risk, while those who had high income for 5 consecutive years had the lowest T2D risk. Those who experienced a greater number of decreases in income had an elevated T2D risk.

**Meaning:**

This cohort study emphasizes the need for public awareness of the role of income fluctuation in T2D risk.

## Teaser

This cohort study investigates whether sustained low or high income and income changes are associated with incidence of type 2 diabetes among adults in South Korea.

## Introduction

The number of people with diabetes is projected to reach 783 million (12.2%) by 2045 worldwide, with middle-income countries experiencing the largest increase in diabetes prevalence by 21%.^1^ Established risk factors of type 2 diabetes (T2D) include obesity and unhealthy lifestyles, including physical inactivity, smoking, poor sleep, and poor diet.^2,3,4^ Socioeconomic status (SES), particularly low income, also contributes to the development of T2D.^5,6,7^ Previous studies have shown that people with low income tend to neglect their health behavior^8,9,10^ and may receive fewer health screenings and health care access than those with high income.^11,12^

Multiple studies have identified positive associations between low income and incidence of T2D.^5,6,13,14,15,16^ However, most studies only used baseline income and did not investigate income status over time with T2D risk. A one-time measurement of income cannot fully reflect the fluctuations in individuals’ income^17^ and may not produce valid estimates for the association between income and T2D risk.^18^ To our knowledge, only a few studies measured income over multiple periods but did not address the patterns and changes in income,^15,16^ had a small number of income categories,^15^ and used self-reported T2D.^15,16^

To fill the evidence gap, we used serial measures of income over the years from the National Health Insurance System (NHIS) in South Korea, which is a universal health insurance coverage system that provides health insurance for 97% of the Korean population. Income status can be identified by the NHIS’s health insurance premiums,^19^ which can be used to assess fluctuations in income. Therefore, we examined the association of low- or high-income status and income changes with incident T2D risk.

## Methods

### Source of Data

The data were derived from the NHIS, a governmental insurer that reviews medical claims for the whole Korean population. Three percent of the Korean people are insured under the Medical Aid Program, which offers health care benefits for those with very-low-income status. The NHIS database comprises information on sociodemographic and medical care, including hospital admission, diagnosis, medical procedures, and prescriptions. It also gathers information on lifestyle characteristics, anthropometric measures, and laboratory tests through national health screening examinations. Further details of the NHIS database are explained elsewhere.^20^ The NHIS database has been validated for research.^21^ This study was approved by the institutional review board of Soongsil University and complies with the ethical guidelines of the World Medical Association Declaration of Helsinki.^22^ This study was conducted in accordance with the Strengthening the Reporting of Observational Studies in Epidemiology (STROBE) reporting guideline.^23^

### Study Population

We initially included 9 498 045 adults aged 30 to 64 years who participated in national health screening examinations in 2012 (baseline year). This age group was regarded as economically active. Individuals who had missing income information (n = 714 625), fasting glucose levels of 126 mg/dL or higher (to convert to millimoles per liter, multiply by 0.0555) (n = 529 558), a history of T2D (n = 254 722), and missing covariates (n = 145 769) at baseline were excluded. We further excluded participants who developed T2D within the first year of follow-up to reduce the bias related to undetected T2D present at baseline (n = 32 144). Finally, a total of 7 821 227 participants were included in this analysis (Figure).

**Figure.**
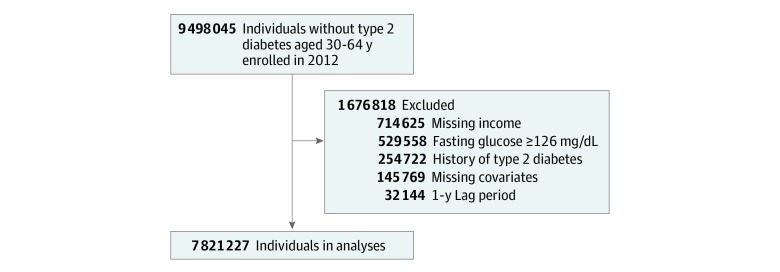
Participant Flow Diagram SI conversion factor: To convert fasting glucose to millimoles per liter, multiply by 0.0555.

### Ascertainment of T2D

Incident T2D was defined if *International Statistical Classification of Diseases and Related Health Problems, Tenth Revision* (*ICD-10*) codes E11 to E14 were present,^20^ if there were 1 or more claims of prescribing antidiabetic medications, or if fasting glucose levels were 126 mg/dL or higher. Those with type 1 diabetes *ICD-10* (code E10) were not included. Incident T2D was identified until December 31, 2019.

### Definition of Income Status

The NHIS does not provide actual household income data. Therefore, we used health insurance premiums as a proxy for household income.^24^ Monthly health insurance premiums are unchanging throughout a 1-year period unless an extreme income change occurs. Wages determine insurance premiums for government and school employees (ie, employee insured) and income and property for the self-employed (ie, self-employed insured).^19^ The monthly health insurance premiums data were presented in 20 quantiles, which were then divided into 4 levels ranging from quarter 1 (lowest income status) to quarter 4 (highest income status). Those who experienced very low income (approximately 3% of the total Korean population) were identified as Medical Aid beneficiaries. When individuals experienced a decrease in income and met the Medical Aid’s criteria, they were eligible to receive Medical Aid the year after the occurrence. Conversely, Medical Aid beneficiaries may transition from Medical Aid to the NHIS if they experience an income increase and meet the requirements.

The number of years a participant was classified in the low- or high-income quartiles was counted annually from 2008 until 2012.^25^ The cumulative number of years of having low or high income was used to examine its association with incidence of T2D. For instance, if a participant experienced low-income status in 2009 and 2011, the score was marked as 2. The relative income percentage changes were calculated across consecutive years: ([income at each time point in 2009, 2010, 2011, and 2012] − [income at 2008, 2009, 2010, and 2011, the 1-year before each time point, *A*])/*A* × 100. An income decrease was ascertained when there was a decrease of 25% or more in the 20 quantiles of health insurance premiums when treated as a continuous variable compared with those in the previous year.^26^ The number of income decreases was categorized as 0, 1, or 2 or more. Additionally, we examined the changes in income quartile using the information on income change between 2008 (the first time point) and 2012 (the last time point at enrollment). Covariates included clinical and anthropometric variables (blood pressure, fasting blood glucose level, triglycerides level, and high-density lipoprotein cholesterol level, body mass index, and waist circumference) and lifestyle-related characteristics (alcohol consumption, smoking status, and physical activity) (see eMethods in Supplement 1 for details).

### Statistical Analysis

Baseline characteristics were presented as mean (SD) values or numbers (percentages). The T2D incidence rate was calculated by dividing the number of events by the total number of person-years of follow-up and exhibited per 1000 person-years. Person-time was calculated from the age of 1 year after baseline until the age at T2D diagnosis or until death, or last follow-up, whichever occurred first. Cox proportional hazards models were used to estimate hazard ratios (HRs) and 95% CIs for the association between variables of income dynamics and T2D risk. The proportional hazards assumption was assessed using the Schoenfeld residuals test with the logarithm of the cumulative hazards function based on Kaplan-Meier estimates for various income parameters. No significant departure from proportionality in hazards over time was observed. The *P* value for a linear trend was estimated using a linear regression model, assigning the ordinal number appointed to each category of income parameters.

We adjusted for potential confounders at enrollment using 3 sequential models. In addition to an unadjusted model (model 1), model 2 was adjusted for age (continuous), sex, and residential location. Model 3 was further adjusted for metabolic parameters (high fasting glucose level, high blood pressure, high triglycerides level, and low level of high-density lipoprotein cholesterol). We did not include obesity and lifestyle characteristics (ie, smoking, alcohol consumption, and physical activity) in the main models because of their potential mediating roles in the association between income status and T2D risk.^27,28^ We assessed the potential effect modification by age group, sex, obesity, current smoking, heavy drinking, regular exercise, health insurance type, and high fasting glucose level using stratified analyses and tests for corresponding interaction terms based on likelihood ratio tests. We conducted the following sensitivity analyses: (1) we further adjusted for smoking status, alcohol consumption, physical activity, body mass index, and waist circumference (model 4) and for 20 income quantiles in the year 2008 (model 5) to evaluate whether the results are robust to the inclusion of the additional covariates. We also further adjusted for the number of income increases in the association between the number of income decreases and T2D risk to consider the potential income fluctuation (model 6); (2) we applied landmark analysis with a 5-year landmark point to estimate the T2D risk for individuals who were event free until a 5-year follow-up and examined the long-term association between variables of income dynamics and T2D risk^29^; and (3) we excluded those who had a history of cancer and cardiovascular disease that might be associated with the decrease in income over time periods before baseline.^30^ Statistical analyses were performed using SAS, version 9.4 (SAS Institute Inc). The *P* values provided are 2-sided, with the level of statistical significance at .05.

## Results

Baseline characteristics are shown in Table 1 and eTables 1 to 2 in Supplement 1. A total of 7 821 227 participants (mean [SD] age, 46.4 [9.3] years; 54.9% men; 78.0% insured by their employer and 1.2% receiving Medical Aid) were included in the analyses. Individuals who consecutively had low income (for 5 years [n = 501 554]) were more likely to be female (65.7%), older (≥45 years [72.4%]), rural residents (10.1%), never smokers (71.0%), nondrinkers (62.9%), inactive (50.8%), and underweight (3.6%), were less likely to be self-employed (14.7%), and had a low level of high-density lipoprotein cholesterol compared with those who never had low income (0 years [32.1% vs 25.4%]). This similar pattern was observed in the number of income decreases. Those who consecutively had high income tended to exhibit opposite trends overall compared with the group who never had high income.

**Table 1.** Characteristics at Baseline by Cumulative Number of Years of Being in Low- and High-Income Status and the Number of Income Decreases

Baseline characteristic	Total	Cumulative No. of years of being in low-income quartile^a^			Cumulative No. of years of being in high-income quartile^a^			No. of income decreases		
		0	1-4	5	0	1-4	5	0	1	≥2
No.	7 821 227	5 218 630	2 101 043	501 554	3 529 516	2 194 409	2 097 302	6 187 532	1 493 834	139 861
Categorical variables, %										
Sex										
Male	54.9	62.6	40.7	34.3	49.1	53.3	66.2	58.4	41.7	38.1
Female	45.1	37.4	59.3	65.7	50.9	46.7	33.8	41.6	58.3	61.9
Age group, y										
<45	45.0	47.3	43.4	27.6	46.9	49.1	37.4	45.7	42.3	40.9
45 to <55	33.4	32.9	33.0	40.2	31.1	28.7	42.2	33.5	32.9	33.5
≥55	21.6	19.8	23.7	32.2	22.0	22.2	20.3	20.8	24.8	25.6
Health insurance type										
Self-employed insured	20.7	22.1	18.7	14.7	18.4	23.6	21.7	21.7	17.6	12.1
Employee insured	78.0	77.9	80.4	70.0	79.0	76.3	78.3	77.1	81.0	86.0
Medical Aid	1.2	NA	0.9	15.3	2.6	0.1	NA	1.2	1.4	1.9
Residential location										
Metropolitan	38.5	39.8	36.5	33.3	35.1	40.1	42.5	38.6	37.9	38.9
Urban	53.5	52.8	54.7	56.6	55.7	52.3	51.1	53.5	53.7	52.8
Rural	8.0	7.5	8.8	10.1	9.2	7.7	6.4	7.9	8.4	8.3
Baseline income										
Medical Aid	1.2	NA	0.9	15.3	2.6	0.1	NA	1.2	1.5	1.9
Quartile 1	16.3	NA	40.3	84.7	26.9	14.7	NA	8.9	42.3	63.2
Quartile 2	19.9	15.9	34.5	NA	34.9	14.7	NA	16.9	31.8	25.8
Quartile 3	27.7	34.6	17.2	NA	35.6	37.7	3.9	30.8	16.9	6.6
Quartile 4	35.0	49.5	7.1	NA	NA	32.7	96.1	42.3	7.5	2.5
Smoking										
Never	57.9	53.3	66.2	71.0	60.1	59.1	52.9	55.7	65.9	68.3
Former	16.1	18.9	10.8	9.5	12.6	15.2	22.9	17.3	11.5	10.7
Current	26.0	27.8	23.0	19.5	27.3	25.6	24.2	26.9	22.6	21.0
Alcohol consumption, g/d										
None	48.5	44.7	54.6	62.9	51.3	49.0	43.4	46.9	54.7	56.4
Mild to moderate (<30)	43.9	47.0	39.1	31.9	41.5	43.6	48.4	45.2	39.1	37.7
Heavy (≥30)	7.6	8.3	6.3	5.2	7.3	7.4	8.2	7.9	6.3	5.9
Physical activity										
None	43.5	40.6	49.2	50.8	47.9	44.6	35.1	42.2	48.6	50.0
Nonregular	37.2	39.5	33.2	30.3	34.6	37.0	42.0	38.3	33.5	32.4
Regular^b^	19.2	19.9	17.6	18.9	17.5	18.5	22.9	19.5	17.9	17.6
BMI										
<18.5	3.0	2.8	3.4	3.6	3.5	2.9	2.1	2.9	3.2	3.2
18.5 to <23	39.1	37.9	41.7	40.9	40.8	39.2	36.3	38.5	41.4	41.7
23 to <25	25.2	25.7	24.0	24.0	23.9	25.0	27.4	25.4	24.2	24.1
25 to <30	29.2	30.1	24.1	27.6	27.9	29.3	31.2	29.6	27.5	27.3
≥30	3.6	3.5	3.8	3.9	4.0	3.6	2.9	3.6	3.7	3.7
High waist circumference^c^	17.8	18.0	17.0	18.7	17.4	18.0	18.1	17.9	17.2	17.3
High BP^c^	39.6	39.6	38.6	43.0	40.1	38.5	39.8	39.8	38.6	37.9
High fasting glucose^c^	25.5	25.8	24.5	26.4	25.4	24.6	26.7	25.8	24.6	24.2
High triglycerides^c^	32.4	33.7	29.4	32.0	31.4	32.0	34.6	33.2	29.7	28.8
Low HDL-C^c^	26.3	25.4	27.2	32.1	26.3	26.4	26.4	26.0	27.5	27.8
Continuous variables, mean (SD)										
Age, y	46.4 (9.3)	45.9 (9.1)	46.7 (9.6)	50.1 (8.4)	45.9 (9.7)	45.8 (9.8)	47.8 (7.7)	46.2 (9.2)	47.0 (9.6)	47.2 (9.6)
BMI	23.8 (3.2)	23.8 (3.1)	23.6 (3.3)	23.7 (3.3)	23.7 (3.3)	23.8 (3.2)	23.9 (2.9)	23.8 (3.2)	23.6 (3.2)	23.6 (3.2)
Waist circumference, cm	80.0 (9.0)	80.5 (8.8)	78.8 (9.1)	78.9 (9.1)	79.3 (9.1)	79.9 (9.0)	81.0 (8.5)	80.2 (8.9)	79.0 (9.0)	78.8 (9.1)
Systolic BP, mm Hg	120.9 (14.0)	121.0 (14.0)	120.6 (14.0)	121.3 (15.0)	121.2 (14.0)	120.7 (14.0)	120.8 (14.0)	121.0 (14.0)	120.5 (14.0)	120.2 (14.0)
Diastolic BP, mm Hg	76.0 (10.0)	76.2 (9.9)	75.7 (10.0)	76.0 (10.2)	76.1 (10.0)	75.8 (9.9)	76.2 (9.9)	76.2 (9.9)	75.6 (10.0)	75.4 (10.0)
Fasting glucose, mg/dL	93.2 (10.9)	93.4 (10.8)	92.8 (11.0)	93.4 (11.2)	93.0 (11.1)	93.0 (10.8)	93.8 (10.9)	93.3 (10.9)	92.9 (10.9)	92.8 (10.9)
Triglycerides, median (IQR), mg/dL	109.4 (109.4-109.5)	112.0 (112.0-112.1)	104.1 (104.0-104.2)	105.9 (105.7-106.0)	107.8 (107.7-107.8)	108.7 (108.6-108.8)	113.1 (113.1-113.2)	110.9 (110.8-110.9)	104.3 (104.2-104.4)	102.9 (102.6-103.2)
HDL-C, mg/dL	55.8 (17.6)	55.1 (17.4)	57.2 (18.1)	57.0 (17.4)	56.6 (18.1)	55.8 (17.5)	54.4 (16.8)	55.5 (17.6)	57.0 (17.8)	57.2 (17.8)

Abbreviations: BMI, body mass index (calculated as weight in kilograms divided by height in meters squared); BP, blood pressure; HDL-C, high-density lipoprotein cholesterol; NA, not applicable.

SI conversion factor: To convert fasting glucose to millimoles per liter, multiply by 0.0555; HDL-C to millimoles per liter, multiply by 0.0259; and triglycerides to millimoles per liter, multiply by 0.0113.

^a^ The number of times an individual was categorized in the low- or high-income quartile was counted every year from 2008 to 2012 (baseline year).

^b^ Regular exercise was defined to be at least 30 minutes of moderate physical activity per day at least 5 days a week or at least 20 minutes of strenuous physical activity per day at least 3 days a week.

^c^ Abnormal metabolic status was defined as following: high waist circumference (≥90 cm for men and ≥85 cm for women), high BP (systolic, ≥130 mm Hg; diastolic, ≥80 mm Hg; or use of antihypertensive medication), high fasting glucose level (≥100 mg/dL), high triglycerides level (≥150 mg/dL or use of a relevant medication), and low HDL-C level (<40 mg/dL for men and <50 mg/dL for women or use of a relevant medication).

Associations of cumulative income status and the number of income decreases with incident T2D risk are displayed in Table 2. There was a total of 359 931 incident T2D cases (4.6% of study participants) at least 1 year after enrollment during a median follow-up of 6.3 years (IQR, 6.1-6.6 years). The cumulative number of years of low- and very-low-income status was associated with increased T2D risk after adjusting for potential confounders in model 3 (1 year vs 0 years: HR, 1.09 [95% CI, 1.08-1.10]; 2 years vs 0 years: HR, 1.10 [95% CI, 1.09-1.12]; 3 years vs 0 years: HR, 1.13 [95% CI, 1.11-1.14]; 4 years vs 0 years: HR, 1.14 [95% CI, 1.12-1.16]; 5 years vs 0 years: HR, 1.22 [95% CI, 1.21-1.23]; *P* < .001 for trend for cumulative number of years of low-income status; 1 year vs 0 years: HR, 1.47 [95% CI, 1.40-1.55]; 2 years vs 0 years: HR, 1.48 [95% CI, 1.41-1.56]; 3 years vs 0 years: HR, 1.52 [95% CI, 1.44-1.61]; 4 years vs 0 years: HR, 1.50 [95% CI, 1.41-1.59]; 5 years vs 0 years: HR, 1.57 [95% CI, 1.53-1.62]; *P* < .001 for trend for cumulative number of years of very-low-income status). Individuals who were consecutively in the low-income quartile (5 years) had the highest T2D risk (model 3: HR, 1.22 [95% CI, 1.21-1.23]) compared with those who never had low income. This association was stronger for those who consecutively had very low income (model 3: HR, 1.57 [95% CI, 1.53-1.62]) compared with those who never had very low income. In addition, just 1 year of having very low income was associated with a 47% increased T2D risk (95% CI, 1.40-1.55). In contrast, those who were consecutively in the high-income quartile had the lowest risk (model 3: HR, 0.86 [95% CI, 0.85-0.86]) compared with those who never had high income. The number of income decreases was associated with increased T2D risk (≥2 vs 0 income decreases in model 3: HR, 1.08 [95% CI, 1.06-1.11]; *P* < .001 for trend). In sensitivity analyses, after further adjusting for potential mediators, including lifestyles and obesity parameters, and income in 2008, the associations were attenuated, particularly for those who consecutively had very low income (relative changes of HRs, ranging from −11.6% to −15.6%) but exhibited similar trends overall (eTable 3 in Supplement 1). The positive associations between the number of income decreases and T2D risk were not changed after further adjustment for the number of income increases (eTable 4 in Supplement 1).

**Table 2.** Associations of Cumulative Income Status and the Number of Income Decreases With Risk of Incident Type 2 Diabetes

Variable	No. of participants	No. of events	Total No. of person-years of follow-up	Incidence rate, per 1000 person-years	Hazard ratio (95% CI)		
					Model 1^a^	Model 2^b^	Model 3^c^
Cumulative No. of years of being in low-income quartile^d^							
0	5 218 630	230 469	32 498 913	7.1	1 [Reference]	1 [Reference]	1 [Reference]
1	835 652	37 955	5 194 246	7.3	1.03 (1.02-1.04)	1.09 (1.08-1.10)	1.09 (1.08-1.10)
2	546 814	25 274	3 398 630	7.4	1.05 (1.04-1.06)	1.10 (1.08-1.11)	1.10 (1.09-1.12)
3	400 853	19 443	2 487 821	7.8	1.10 (1.09-1.12)	1.13 (1.11-1.14)	1.13 (1.11-1.14)
4	317 724	16 351	1 968 269	8.3	1.17 (1.15-1.19)	1.15 (1.13-1.17)	1.14 (1.12-1.16)
5	501 554	30 439	3 086 354	9.9	1.39 (1.38-1.41)	1.26 (1.24-1.27)	1.22 (1.21-1.23)
*P* value for trend					<.001	<.001	<.001
Cumulative No. of years of being in very-low-income status^d^							
0	7 684 335	348 915	47 803 647	7.3	1 [Reference]	1 [Reference]	1 [Reference]
1	20 840	1537	127 291	12.1	1.66 (1.58-1.74)	1.53 (1.46-1.61)	1.47 (1.40-1.55)
2	18 835	1474	114 696	12.9	1.76 (1.68-1.86)	1.60 (1.52-1.69)	1.48 (1.41-1.56)
3	15 090	1145	91 943	12.5	1.71 (1.61-1.81)	1.62 (1.53-1.72)	1.52 (1.44-1.61)
4	15 285	1148	93 003	12.3	1.69 (1.60-1.80)	1.59 (1.50-1.68)	1.50 (1.41-1.59)
5	66 842	5712	403 653	14.2	1.94 (1.89-1.99)	1.68 (1.64-1.73)	1.57 (1.53-1.62)
*P* value for trend					<.001	<.001	<.001
Cumulative No. of years of being in high-income quartile^d^							
0	3 529 516	168 842	21 887 213	7.7	1 [Reference]	1 [Reference]	1 [Reference]
1	714 117	31 777	4 441 779	7.2	0.93 (0.92-0.94)	0.94 (0.93-0.95)	0.95 (0.94-0.96)
2	518 952	22 854	3 231 188	7.1	0.92 (0.90-0.93)	0.92 (0.91-0.93)	0.93 (0.92-0.95)
3	484 512	21 403	3 019 181	7.1	0.92 (0.91-0.93)	0.91 (0.89-0.92)	0.93 (0.92-0.92)
4	476 828	20 964	2 970 882	7.1	0.91 (0.90-0.93)	0.88 (0.87-0.89)	0.90 (0.89-0.92)
5	2 097 302	94 091	13 083 988	7.2	0.93 (0.92-0.94)	0.82 (0.82-0.83)	0.86 (0.85-0.86)
*P* value for trend					<.001	<.001	<.001
No. of income decreases							
0	6 187 532	282 471	38 487 586	7.3	1 [Reference]	1 [Reference]	1 [Reference]
1	1 493 834	70 764	9 277 508	7.6	1.00 (1.03-1.05)	1.05 (1.04-1.06)	1.06 (1.05-1.07)
≥2	139 861	6696	869 139	7.7	1.05 (1.03-1.08)	1.06 (1.03-1.09)	1.08 (1.06-1.11)
*P* value for trend					<.001	<.001	<.001

^a^ Unadjusted.

^b^ Adjusted for age (continuous), sex, and residential location (urban, rural, or metropolitan).

^c^ Additionally adjusted for high fasting glucose level (≥100 mg/dL [to convert to millimoles per liter, multiply by 0.0555]), high blood pressure (systolic, ≥130 mm Hg; diastolic, ≥80 mm Hg; or use of antihypertensive medication), high triglycerides level (≥150 mg/dL [to convert to millimoles per liter, multiply by 0.0113] or use of a relevant medication), and low level of high-density lipoprotein cholesterol (<40 mg/dL [to convert to millimoles per liter, multiply by 0.0259] for men and <50 mg/dL for women or use of a relevant medication).

^d^ The number of times an individual was categorized for low-, very-low-, or high-income status was counted every year from 2008 to 2012 (baseline year).

The changes in income status between the 2 time points (2008 and 2012) and the corresponding risk of T2D are presented in Table 3. In each income status in 2008, individuals who experienced an income decrease had an elevated T2D risk, while those who experienced an income increase had lowered T2D risk compared with those who had a persistent income status between 2008 and 2012. For example, those who initially were in the first quartile of income in 2008 had elevated T2D risk when they became Medical Aid beneficiaries (HR, 1.49 [95% CI, 1.40-1.59]) and had lowered risk when their income status increased to the highest quartile (HR, 0.93 [95% CI, 0.90-0.96]). This pattern remained consistent in other income statuses.

**Table 3.** Change of Income Status Between First Time Point in 2008 and Last Time Point at Baseline in 2012, and the Corresponding Risk of Incident Type 2 Diabetes

Income status at baseline in 2012 (last time point)^a^	No. of participants	No. of events	Total No. of person-years of follow-up	Incidence rate, per 1000 person-years	Hazard ratio (95% CI)		
					Model 1^b^	Model 2^c^	Model 3^d^
**First time point in 2008: Medical Aid**							
Medical Aid	68 610	5873	414 187	14.2	1 [Reference]	1 [Reference]	1 [Reference]
Quartile 1	15 349	1249	93 527	13.4	0.94 (0.89-1.00)	0.98 (0.92-1.04)	0.97 (0.91-1.03)
Quartile 2	12 731	795	78 553	10.1	0.71 (0.66-0.77)	0.81 (0.75-0.87)	0.84 (0.78-0.91)
Quartile 3	5662	323	35 045	9.2	0.65 (0.58-0.72)	0.73 (0.65-0.82)	0.78 (0.69-0.87)
Quartile 4	1282	86	7902	10.9	0.76 (0.62-0.95)	0.81 (0.65-0.99)	0.83 (0.67-1.03)
*P* value for trend					<.001	<.001	<.001
**First time point in 2008: quartile 1**							
Medical Aid	11 312	1018	67 747	15.0	1.74 (1.64-1.86)	1.62 (1.52-1.72)	1.49 (1.40-1.59)
Quartile 1	593 151	31 725	3 669 117	8.6	1 [Reference]	1 [Reference]	1 [Reference]
Quartile 2	399 238	17 513	2 481 276	7.1	0.82 (0.80-0.83)	0.97 (0.95-0.99)	0.98 (0.97-1.00)
Quartile 3	196 091	8757	1 220 211	7.2	0.83 (0.81-0.85)	0.97 (0.95-1.00)	0.97 (0.94-0.99)
Quartile 4	77 532	3889	482 288	8.1	0.93 (0.90-0.96)	0.93 (0.90-0.96)	0.93 (0.90-0.96)
*P* value for trend					<.001	<.001	<.001
**First time point in 2008: quartile 2**							
Medical Aid	9427	748	57 176	13.1	1.77 (1.64-1.90)	1.61 (1.49-1.73)	1.52 (1.42-1.64)
Quartile 1	265 505	13 617	1 644 308	8.3	1.12 (1.09-1.14)	1.04 (1.02-1.07)	1.05 (1.03-1.07)
Quartile 2	690 720	31 760	4 283 826	7.4	1 [Reference]	1 [Reference]	1 [Reference]
Quartile 3	621 216	25 484	3 869 341	6.6	0.89 (0.87-0.90)	0.99 (0.98-1.02)	0.99 (0.97-1.00)
Quartile 4	112 740	5395	701 654	7.7	1.03 (1.01-1.07)	0.98 (0.95-1.01)	0.98 (0.95-1.01)
*P* value for trend					<.001	<.001	<.001
**First time point in 2008: quartile 3**							
Medical Aid	4436	332	26 986	12.3	1.73 (1.55-1.93)	1.59 (1.42-1.77)	1.53 (1.38-1.71)
Quartile 1	220 302	10 618	1 367 752	7.8	1.09 (1.07-1.11)	1.04 (1.02-1.06)	1.07 (1.05-1.09)
Quartile 2	283 018	13 376	1 755 680	7.6	1.07 (1.05-1.09)	1.02 (1.00-1.04)	1.04 (1.02-1.06)
Quartile 3	1 100 326	48 730	6 841 758	7.1	1 [Reference]	1 [Reference]	1 [Reference]
Quartile 4	613 382	24 123	3 832 332	6.3	0.88 (0.87-0.90)	0.95 (0.94-0.97)	0.97 (0.96-0.99)
*P* value for trend					<.001	<.001	<.001
**First time point in 2008: quartile 4**							
Medical Aid	1622	125	9878	12.7	1.74 (1.46-2.08)	1.74 (1.46-2.07)	1.69 (1.42-2.02)
Quartile 1	176 731	7952	1 099 838	7.2	1.00 (0.97-1.02)	1.06 (1.04-1.09)	1.09 (1.07-1.12)
Quartile 2	170 248	7633	1 058 881	7.2	0.99 (0.97-1.02)	1.07 (1.04-1.09)	1.08 (1.05-1.11)
Quartile 3	241 599	11 266	1 501 523	7.5	1.03 (1.01-1.05)	1.06 (1.03-1.08)	1.04 (1.02-1.06)
Quartile 4	1 928 997	87 544	12 033 445	7.3	1 [Reference]	1 [Reference]	1 [Reference]
*P* value for trend					.56	<.001	<.001

^a^ Quartile 1 is the lowest income status, and quartile 4 is the highest income status.

^b^ Unadjusted.

^c^ Adjusted for age (continuous), sex, and residential location (urban, rural, or metropolitan).

^d^ Additionally adjusted for high fasting glucose level (≥100 mg/dL [to convert to millimoles per liter, multiply by 0.0555]), high blood pressure (systolic, ≥130; diastolic, ≥80 mm Hg; or use of antihypertensive medication), high triglycerides level (≥150 mg/dL [to convert to millimoles per liter, multiply by 0.0113] or use of a relevant medication), and low level of high-density lipoprotein cholesterol (<40 mg/dL [to convert to millimoles per liter, multiply by 0.0259] for men and <50 mg/dL for women or use of a relevant medication).

Associations of cumulative income status and the number of income decreases with T2D risk by potential effect modifiers are shown in Table 4. The association between more income decreases (≥2 vs 0) and elevated T2D risk was observed only for those who were older (≥45 years) (45-54 years: adjusted HR, 1.15 [95% CI, 1.11-1.20]; ≥55 years: adjusted HR, 1.07 [95% CI, 1.03-1.11]). The association between 5 consecutive years in the high-income quartile (5 vs 0) and lowered T2D risk was stronger for those who led a healthy lifestyle (nonsmoker: adjusted HR, 0.85 [95% CI, 0.84-0.86]; nondrinker: adjusted HR, 0.84 [95% CI, 0.83-0.85]; exercised regularly, adjusted HR, 0.84 [95% CI, 0.83-0.86]). Additionally, the association between more income decreases (≥2 vs 0) and elevated T2D risk was stronger for those who did not exercise regularly (adjusted HR, 1.10 [95% CI, 1.07-1.13]). In our sensitivity analyses, the results did not substantially differ after excluding those with a history of cancer and cardiovascular disease (eTable 5 in Supplement 1), nor when our analysis was limited to individuals who were event free until the fifth year of follow-up (eTable 6 in Supplement 1).

**Table 4.** Associations of Cumulative Income Status and Number of Income Decreases With Risk of Incident Type 2 Diabetes by Selected Factors

Factor	Cumulative No. of years of being in low-income quartile (5 vs 0)		Cumulative No. of years of being in high-income quartile (5 vs 0)		No. of income decreases (≥2 vs 0)	
	Adjusted HR (95% CI)^a^	*P* value for interaction	Adjusted HR (95% CI)^a^	*P* value for interaction	Adjusted HR (95% CI)^a^	*P* value for interaction
Age group, y						
<45	1.22 (1.19-1.27)	.02	0.91 (0.89-0.92)	<.001	0.99 (0.95-1.06)	<.001
45-54	1.24 (1.22-1.27)		0.83 (0.82-0.84)		1.15 (1.11-1.20)	
≥55	1.20 (1.18-1.22)		0.83 (0.82-0.84)		1.07 (1.03-1.11)	
Sex						
Male	1.26 (1.24-1.28)	<.001	0.86 (0.85-0.87)	.001	1.08 (1.04-1.12)	.98
Female	1.19 (1.17-1.21)		0.84 (0.83-0.85)		1.08 (1.05-1.12)	
Current smoking						
No	1.20 (1.18-1.22)	.32	0.85 (0.84-0.86)	<.001	1.09 (1.06-1.12)	.07
Yes	1.18 (1.16-1.21)		0.95 (0.94-0.96)		1.03 (0.99-1.09)	
Heavy drinking						
No	1.22 (1.20-1.24)	.65	0.84 (0.83-0.85)	<.001	1.10 (1.07-1.14)	.08
Yes	1.21 (1.19-1.24)		0.88 (0.87-0.89)		1.05 (1.01-1.09)	
Regular exercise						
No	1.22 (1.21-1.24)	.51	0.86 (0.85-0.87)	.01	1.10 (1.07-1.13)	.01
Yes	1.21 (1.18-1.24)		0.84 (0.83-0.86)		1.01 (0.95-1.07)	
Health insurance type						
Self-employed insured	1.20 (1.17-1.23)	.004	0.87 (0.86-0.88)	.45	1.14 (1.06-1.21)	.40
Employee insured	1.14 (1.13-1.16)		0.86 (0.85-0.87)		1.09 (1.06-1.12)	
Medical Aid	NA		NA		1.04 (0.90-1.19)	
Obesity (BMI ≥25)						
No	1.30 (1.28-1.33)	<.001	0.87 (0.86-0.89)	<.001	1.09 (1.05-1.13)	.66
Yes	1.18 (1.16-1.20)		0.83 (0.82-0.84)		1.07 (1.04-1.11)	
High fasting glucose level						
No	1.39 (1.36-1.41)	<.001	0.74 (0.73-0.75)	<.001	1.09 (1.04-1.13)	.85
Yes	1.13 (1.11-1.15)		0.93 (0.92-0.94)		1.08 (1.05-1.11)	

Abbreviations: BMI, body mass index (calculated as weight in kilograms divided by height in meters squared); HR, hazard ratio; NA, not applicable.

^a^ Adjusted for age (continuous), sex, and residential location (urban, rural, or metropolitan), high fasting glucose level (≥100 mg/dL [to convert to millimoles per liter, multiply by 0.0555]), high blood pressure (systolic, ≥130 mm Hg; diastolic, ≥80 mm Hg; or use of antihypertensive medication), high triglycerides level (≥150 mg/dL [to convert to millimoles per liter, multiply by 0.0113] or use of a relevant medication), and low level of high-density lipoprotein cholesterol (<40 mg/dL [to convert to millimoles per liter, multiply by 0.0259] for men and <50 mg/dL for women or use of a relevant medication).

## Discussion

In this nationwide population-based cohort study of more than 7.8 million participants, sustained low-income status and an income decrease were associated with elevated T2D risk. Individuals who consecutively had low income for the past 5 years had a 22% elevated T2D risk compared with those who never had low income. Additionally, individuals who repeatedly had very low income (Medical Aid beneficiaries) had a 57% higher T2D risk compared with those who never had very low income. Furthermore, a greater number of income decreases over 5 years was associated with elevated T2D risk. Those who experienced an income increase had a lowered T2D risk, independent of the individual’s initial income status. This is the first study to investigate the association of sustained low and high income and income changes with T2D development, to our knowledge.

Multiple studies have investigated the association between low income and T2D incidence. A meta-analysis of 5 studies published until 2010 found that people in the lowest income level had a 40% higher T2D risk compared with those in the highest income level.^5^ A few more studies provided further evidence of this association based on various indicators of income, including annual household and neighborhood income.^6,13,14^ These studies presented an overall inverse association between income and T2D risk. However, income was measured at a single point in time and does not reflect income dynamics. Furthermore, there is little evidence of the role of changing SES in the development of T2D. While only a few studies observed income from multiple time points,^15,16^ they did not consider the roles of patterns and changes in income, which were addressed in our study. There were also few studies that investigated the association between overall SES and T2D incidence at multiple time points.^5^ The Nurses’ Health Study assessed SES changes from childhood to adulthood, finding that women with a decreasing SES had an 18% higher T2D risk compared with those who had a stable SES.^31^

Our study found significant positive associations between an income decrease and elevated T2D risk, which were stronger for a greater number of income decreases. Prior studies reported similar results with different outcomes. In the Coronary Artery Risk Development in Young Adults study, a greater number of income decreases was associated with deteriorated brain function^32^ and with elevated risk of cardiovascular disease and all-cause mortality.^26^ Pool et al^33^ reported that a dramatic decrease in wealth was associated with elevated all-cause mortality.

We also found that individuals with sustained high income or those who experienced an income increase had lowered T2D risk. We previously found that an income increase was associated with lowered mortality risk for patients with T2D,^25^ and an Australian study reported that children in families experiencing an income increase had a 60% lowered risk of asthma.^18^ However, there is little evidence on the association between individuals’ income increase and risk of developing T2D. Instead, a few studies examined the association between neighborhood SES and T2D risk. A Spanish study reported that improving neighborhood SES was associated with decreased T2D risk.^34^ A randomized social experiment study showed that transitioning from high to low status at the neighborhood level was associated with decreased prevalence of diabetes.^35^ However, these studies did not consider individuals’ SES change.

Several mechanisms have been suggested to understand the association between low income and T2D risk. People with low income tend to develop unhealthy habits due to stress from their financial struggles.^9^ Depending on the degree of economic change, the more financial trouble individuals faced, the more likely it was for them to spur on harmful habits, such as smoking and physical inactivity.^10,36^ We evaluated the role of lifestyle factors in a sensitivity analysis (model 4), finding that the association was attenuated particularly for those with very low income for all 5 years. This finding suggests that lifestyle factors may influence this association for individuals in this income status more than others. Low income was also associated with decreased access to health care, and being unable to seek regular health screenings and proper treatments,^12^ which may lead to preventable health complications.^37^ Additionally, low income was associated with food insecurity and poor diet quality,^38^ leading to increased T2D risk.^39^ Food insecurity was associated with the consumption of foods that are high in calories and fat, which can be a cheaper option than nutritional foods high in fiber.^40^ Moreover, inflammation may influence the association between low SES and adverse health outcomes,^41,42^ although the role of C-reactive protein was not confirmed in the association between low-income–related behavior and T2D risk.^41^

### Limitations and Strengths

This study has some limitations worth noting. First, data on actual household income were unavailable. Additionally, we used health insurance premiums assessed at the end of each year, which may not capture the income change during that year. Second, the follow-up duration was relatively short. Third, we could not address a specific community with a greater socioeconomic diversity because this study was based on secondary administrative data. Fourth, our findings may not apply to populations with different cultural and economic backgrounds. Lastly, information on potential confounders, such as a family history of diabetes and dietary intake, was unavailable. Despite these limitations, this study was based on a large sample size of more than 7.8 million individuals, enabling us to evaluate various sensitivity analyses and effect modifications by demographic and lifestyle factors. Furthermore, our findings suggest the importance of income inequality as a social determinant of health that could influence the risk of T2D, particularly among nonelderly individuals in terms of poverty and limited access to health care services and resources in a middle-income Asian country.^43^

## Conclusions

This cohort study found that, for more than 7.8 million Korean adults, sustained low income and an income decrease were associated with elevated T2D risk, whereas sustained high income was associated with lowered T2D risk. Our study provides insight into the association of the pattern and changes in income with the development of T2D. Future studies are warranted to clarify the mechanisms underlying the association of sustained low income and an income decrease with T2D risk.

## Notes

Supplement 1. **eMethods.** Measurments of Covariates
eReferences.
**eTable 1.** Characteristics at Baseline by Cumulative Number of Years of Being in Low-Income Status **eTable 2.** Characteristics at Baseline by Cumulative Number of Years of Being in High-Income Status **eTable 3.** Association of Cumulative Income Status and the Number of Income Decreases With Risk of Incident Type 2 Diabetes Further Adjusted for Lifestyle Factors, Obesity, and Income in 2008 **eTable 4.** Association of the Number of Income Decreases With Risk of Incident Type 2 Diabetes Further Adjusted for the Number of Income Increases **eTable 5.** Association of Cumulative Income Status and the Number of Income Decreases With Risk of Incident Type 2 Diabetes, After Excluding Those With a Prior History of Cancer and Cardiovascular Disease **eTable 6.** Five-Year Landmark Analysis on the Association of Cumulative Income Status and the Number of Income Decreases With Risk of Incident Type 2 Diabetes

Supplement 2.
Data Sharing Statement
