# Supplementary material for: Association of Sustained Low or High Income and Income Changes With Risk of Incident Type 2 Diabetes Among Individuals Aged 30 to 64 Years
Source: JAMA Netw Open. 2023 Aug 21;6(8):e2330024. doi: 10.1001/jamanetworkopen.2023.30024 (PMC10442710; doi:10.1001/jamanetworkopen.2023.30024)
Supplement: Supplement 1. — eMethods. Measurments of Covariates eReferences. eTable 1. Characteristics at Baseline by Cumulative Number of Years of Being in Low-Income Status eTable 2. Characteristics at Baseline by Cumulative Number of Years of Being in High-Income Status eTable 3. Association of Cumulative Income Status and the Number of Income Decreases With Risk of Incident Type 2 Diabetes Further Adjusted for Lifestyle Factors, Obesity, and Income in 2008 eTable 4. Association of the Number of Income Decreases With Risk of Incident Type 2 Diabetes Further Adjusted for the Number of Income Increases eTable 5. Association of Cumulative Income Status and the Number of Income Decreases With Risk of Incident Type 2 Diabetes, After Excluding Those With a Prior History of Cancer and Cardiovascular Disease eTable 6. Five-Year Landmark Analysis on the Association of Cumulative Income Status and the Number of Income Decreases With Risk of Incident Type 2 Diabetes [file jamanetwopen-e2330024-s001.pdf]

Supplemental Online Content

Park JC, Nam GE, Yu J, et al. Association of sustained low or high income and income changes with risk of incident type 2 diabetes among individuals aged 30 to 64 years. *JAMA Netw Open*. 2012;6(8):e2330024. doi:10.1001/jamanetworkopen.2023.30024

eMethods. Measurements of Covariates

eReferences.

eTable 1. Characteristics at Baseline by Cumulative Number of Years of Being in Low-Income Status

eTable 2. Characteristics at Baseline by Cumulative Number of Years of Being in High-Income Status

eTable 3. Association of Cumulative Income Status and the Number of Income Decreases With Risk of Incident Type 2 Diabetes Further Adjusted for Lifestyle Factors, Obesity, and Income in 2008

eTable 4. Association of the Number of Income Decreases With Risk of Incident Type 2 Diabetes Further Adjusted for the Number of Income Increases

eTable 5. Association of Cumulative Income Status and the Number of Income Decreases With Risk of Incident Type 2 Diabetes, After Excluding Those With a Prior History of Cancer and Cardiovascular Disease

eTable 6. Five-Year Landmark Analysis on the Association of Cumulative Income Status and the Number of Income Decreases With Risk of Incident Type 2 Diabetes

This supplemental material has been provided by the authors to give readers additional information about their work.

© 2023 Park JC et al. *JAMA Network Open.*

eMethods.

Measurements of covariates

Clinical and anthropometric variables were measured by trained personnel during the national health screening examinations. Blood pressure (BP) was measured once the individual was in a resting sitting position for at least 5 minutes. High BP was defined as systolic BP ≥130 mmHg or diastolic BP ≥80 mmHg or the current use of antihypertensive medication. After an overnight fasting of at least 8 hours, blood samples were collected, and serum levels of glucose, triglycerides, and high-density lipoprotein cholesterol (HDL-C) were measured. High fasting glucose was defined as ≥100 mg/dL; high triglycerides levels were defined as ≥150 mg/dL or the use of a relevant medication; low HDL-C levels were defined as <40 mg/dL for men and <50 mg/dL for women or the use of a relevant medication.[1] Body mass index (BMI) was calculated by the division of the weight (kg) by the square of height (kg/m^2). We categorized individuals into underweight (<18.5 kg/m^2), normal (18.5–22.9 kg/m^2), overweight (23–24.9 kg/m^2), obese (25–29.9 kg/m^2), or severely obese (≥30) kg/m^2 groups based on World Health Organization recommendations for Asian people.[2] Based on Korean population standards, abdominal obesity was defined as having a waist circumference of ≥90 cm for men and ≥85 cm for women.[3]

Lifestyle-related characteristics were measured using self-administered questionnaires. Alcohol consumption was classified into nondrinking, mild to moderate drinking (<30 g/day), and heavy drinking (≥30 g/day). Smoking status was categorized as never, former, or current smoker. Physical activity was categorized as regular (vigorous exercise ≥3 times per week or moderate-intensity exercise ≥5 times per week), non-regular, or no physical activity.[4]

© 2023 Park JC et al. *JAMA Network Open.*

eReferences.

1. Alberti KG, Eckel RH, Grundy SM, et al. Harmonizing the metabolic syndrome: a joint interim statement of the international diabetes federation task force on epidemiology and prevention; national heart, lung, and blood institute; American heart association; world heart federation; international atherosclerosis society; and international association for the study of obesity. *Circulation*. 2009;120(16):1640-1645.
2. Organization WH. International association for the study of obesity, International Obesity Taskforce. *The Asia-Pacific perspective: redefining obesity and its treatment*. 2000:15-21.
3. Seo MH, Lee WY, Kim SS, et al. 2018 Korean Society for the Study of Obesity Guideline for the Management of Obesity in Korea. *J Obes Metab Syndr*. Mar 2019;28(1):40-45. doi:10.7570/jomes.2019.28.1.40
4. Chun MY. Validity and reliability of korean version of international physical activity questionnaire short form in the elderly. *Korean J Fam Med*. May 2012;33(3):144-51. doi:10.4082/kjfm.2012.33.3.144

© 2023 Park JC et al. *JAMA Network Open.*

eTable 1. Characteristics at baseline by cumulative number of years of being in low-income status

Baseline characteristics	Cumulative no. of years of being in low-income quartile[a]					
	**0**	**1**	**2**	**3**	**4**	**5**
N	5,218,630	835,652	546,814	400,853	317,724	501,554
**Categorical variables**						
Sex, %						
Male	62.6	46.6	39.8	35.7	33.3	34.3
Female	37.4	53.4	60.2	64.3	66.7	65.7
Age group (years), %						
<45	47.3	47.2	44.0	40.5	35.9	27.6
45-<55	32.9	30.9	32.9	34.8	36.3	40.2
≥55	19.8	21.9	23.1	24.8	27.9	32.2
Health insurance type, %		46.6	39.8	35.7	33.3	
Self-employed insured	22.1	20.2	18.7	17.3	16.7	14.7
Employee insured	77.9	79.5	80.7	81.2	81.2	70.0
Medical Aid	NA	0.3	0.6	1.5	2.2	15.3
Residential location, %						
Metropolitan	39.8	37.2	36.5	35.8	35.4	33.3
Urban	52.8	54.2	54.6	55.3	55.6	56.6
Rural	7.5	8.6	8.9	9.0	9.0	10.1
Baseline income, %						
Medical Aid	NA	0.3	0.6	1.5	2.2	15.3
Quartile 1	NA	27.1	38.2	50.7	65.2	84.7
Quartile 2	15.9	36.5	38.4	33.4	24.3	NA
Quartile 3	34.6	25.5	16.3	10.2	5.9	NA
Quartile 4	49.5	10.6	6.5	4.2	2.4	NA
Smoking, %						
Never	53.3	62.1	66.9	69.8	71.6	71.0
Former	18.9	12.1	10.4	9.8	9.3	9.5
Current	27.8	25.9	22.7	20.4	19.1	19.5
Alcohol consumption (g/day), %						
None	44.7	51.6	54.8	57.2	59.4	62.9

© 2023 Park JC et al. *JAMA Network Open.*

Mild to moderate (<30)	47.0	41.4	39.0	37.2	35.3	31.9
Heavy (≥30)	8.3	7.0	6.3	5.7	5.3	5.2
Physical activity, %						
None	40.6	48.4	49.5	49.8	49.9	50.8
Non-regular	39.5	34.2	33.1	32.5	31.7	30.3
Regular[b]	19.9	17.3	17.5	17.8	18.4	18.9
Body mass index (kg/m^2), %						
<18.5	2.8	3.3	3.4	3.4	3.3	3.6
18.5-<23	37.9	41.1	42.2	42.1	42.1	40.9
23-<25	25.7	24.0	23.8	24.1	24.1	24.0
25-<30	30.1	27.7	26.8	26.6	26.8	27.6
≥30	3.5	3.9	3.8	3.8	3.7	3.9
High waist circumference[c], %	18.0	17.3	16.8	16.8	17.1	18.7
High BP[c], %	39.6	38.4	38.2	38.7	39.9	43.0
High fasting glucose[c], %	25.8	24.6	24.2	24.5	24.8	26.4
High Triglycerides[c], %	33.7	30.1	29.0	28.8	29.4	32.0
Low HDL-C[c], %	25.4	26.1	27.1	28.2	29.3	32.1
**Continuous variables, mean (SD)**						
Age, years	45.9 (9.1)	45.8 (9.9)	46.5 (9.6)	47.3 (9.4)	48.4 (9.1)	50.1 (8.4)
Body mass index, kg/m^2	23.8 (3.1)	23.7 (3.3)	23.6 (3.3)	23.6 (3.3)	23.6 (3.2)	23.7 (3.3)
Waist circumference, cm	80.5 (8.8)	79.2 (9.1)	78.6 (9.1)	78.4 (9.0)	78.4 (9.0)	78.9 (9.1)
Systolic BP, mmHg	121.0 (14.0)	120.7 (14.3)	120.5 (14.4)	120.5 (14.5)	120.7 (14.6)	121.3 (14.9)
Diastolic BP, mmHg	76.2 (9.9)	75.8 (10.0)	75.6 (10.0)	75.6 (10.0)	75.7 (10.0)	76.0 (10.2)
Fasting glucose, mg/dL	93.4 (10.8)	92.8 (11.0)	92.8 (11.0)	92.8 (11.0)	93.0 (11.02)	93.4 (11.2)
Triglycerides, median (IQR) mg/dL	112.0 (112.0-112.1)	105.7 (105.6-105.8)	103.5 (103.4-103.7)	102.8 (102.6-103.0)	102.8 (102.6-103.0)	105.9 (105.7-106.0)
HDL-C, mg/dL	55.1 (17.4)	56.8 (18.2)	57.3 (18.2)	57.5 (17.8)	57.6 (18.2)	57.0 (17.4)

Data are presented as percentages for categorical variables and means (standard deviation) for continuous variables.
Abbreviations: SD, standard deviation, BP, blood pressure; HDL-C, high-density lipoprotein cholesterol; IQR, interquartile range.
[a] The number of times an individual was categorized in the low- or high-income quartile was counted every year from 2008 to 2012 (baseline year).
[b] Regular exercise was defined to be at least 30 minutes of moderate physical activity per day at least five days a week or at least 20 minutes of strenuous physical activity per day at least three days a week.
[c] Abnormal metabolic status was defined as following: high waist circumference (≥90 cm for men and ≥85 cm for women), high BP (systolic BP ≥130 or diastolic BP ≥80 mmHg or the use of antihypertensive medication), high glucose (≥100 mg/dL), high triglycerides (≥150 mg/dL or the use of a relevant medication), and low HDL-C (<40 mg/dL for men and <50 mg/dL for women or the use of a relevant medication).

© 2023 Park JC et al. *JAMA Network Open.*

eTable2. Characteristics at baseline by cumulative number of years of being in high-income status

Baseline characteristics	Cumulative no. of years of being in high-income quartile[a]					
	0	1	2	3	4	5
N	3,529,516	714,117	518,952	484,512	476,828	2,097,302
**Categorical variables**						
Sex, %						
Male	49.1	52.8	53.0	54.1	53.6	66.2
Female	50.9	47.2	47.0	45.9	46.4	33.8
Age group (years), %						
<45	46.9	50.7	49.6	49.1	46.1	37.4
45-<55	31.1	27.6	28.3	28.5	31.0	42.2
≥55	22.0	21.7	22.1	22.5	22.8	20.3
Health insurance type, %						
Self-employed insured	18.4	20.5	23.3	24.1	27.8	21.7
Employee insured	79.0	79.3	76.6	75.8	72.1	78.3
Medical Aid	2.6	0.2	0.1	0.1	0.1	NA
Residential location, %						
Metropolitan	35.1	38.3	39.7	40.8	42.3	42.5
Urban	55.7	53.7	52.5	51.7	50.6	51.1
Rural	9.2	7.9	7.8	7.6	7.1	6.4
Baseline income, %						
Medical Aid	2.6	0.2	0.1	0.1	0.1	NA
Quartile 1	26.9	18.0	15.5	12.9	10.8	NA
Quartile 2	34.9	20.3	15.7	11.7	8.4	NA
Quartile 3	35.6	47.5	41.4	33.2	23.7	3.9
Quartile 4	NA	14.0	27.2	42.2	57.0	96.1
Smoking, %						
Never	60.11)	58.5	59.1	59.2	60.0	52.9
Former	12.6	14.3	14.9	15.8	16.5	22.9
Current	27.3	27.3	26.0	24.9	23.6	24.2
Alcohol consumption (g/day), %						
None	51.3	48.9	49.1	48.7	49.2	43.4

© 2023 Park JC et al. *JAMA Network Open.*

Mild to moderate (<30)	41.5	43.6	43.5	43.9	43.6	48.4
Heavy (≥30)	7.3	7.6	7.4	7.4	7.2	8.2
Physical activity, %						
None	47.9	45.9	44.9	43.6	43.1	35.1
Non-regular	34.6	36.2	36.8	37.6	37.6	42.0
Regular[b]	17.5	17.9	18.3	18.8	19.3	22.9
Body mass index (kg/m^2), %						
<18.5	3.5	3.1	2.9	2.8	2.6	2.1
18.5-<23	40.8	39.6	39.2	38.9	38.9	36.3
23-<25	23.9	24.6	24.9	25.1	25.6	27.4
25-<30	27.9	29.0	29.2	29.6	29.5	31.2
≥30	4.0	3.8	3.7	3.6	3.4	2.9
High waist circumference[c], %	17.4	17.8	18.1	18.1	18.1	18.1
High BP[c], %	40.1	38.8	38.4	38.3	38.3	39.8
High fasting glucose[c], %	25.4	24.6	24.5	24.4	24.8	26.7
High Triglycerides[c], %	31.4	31.8	31.9	32.2	32.1	34.6
Low HDL-C[c], %	26.3	26.0	26.3	26.5	26.9	26.4
**Continuous variables, mean (SD)**						
Age, years	45.9 (9.7)	45.4 (10.0)	45.7 (9.8)	45.9 (9.7)	46.6 (9.3)	47.8 (7.7)
Body mass index, kg/m^2	23.7 (3.3)	23.8 (3.2)	23.8 (3.2)	23.8 (3.1)	23.8 (3.1)	23.9 (2.9)
Waist circumference, cm	79.3 (9.1)	79.8 (9.0)	79.9 (9.0)	80.0 (9.0)	80.1 (8.9)	81.0 (8.5)
Systolic BP, mmHg	121.2 (14.4)	120.8 (14.2)	120.7 (14.1)	120.6 (14.1)	120.5 (14.1)	120.8 (13.8)
Diastolic BP, mmHg	76.1 (10)	75.9 (9.9)	75.8 (9.9)	75.8 (9.9)	75.7 (9.9)	76.2 (9.9)
Fasting glucose, mg/dL	93.0 (11.1)	92.9 (10.9)	92.9 (10.8)	93.0 (10.7)	93.1 (10.7)	93.8 (10.6)
Triglycerides, median (IQR), mg/dL	107.8 (107.7-107.8)	108.6 (108.4-108.7)	108.7 (108.5-108.8)	108.8 (108.7-109.0)	108.7 (108.5-108.8)	113.1 (113.1-113.2)
HDL-C, mg/dL	56.6 (18.1)	56.0 (17.3)	55.9 (18.1)	55.7 (17.7)	55.6 (17.1)	54.4 (16.8)

Abbreviations: SD, standard deviation; BP, blood pressure; HDL-C, high-density lipoprotein cholesterol; IQR, interquartile range.

[a] The number of times an individual was categorized in the low- or high-income quartile was counted every year from 2008 to 2012 (baseline year).

[b] Regular exercise was defined to be at least 30 minutes of moderate physical activity per day at least five days a week or at least 20 minutes of strenuous physical activity per day at least three days a week.

[c] Abnormal metabolic status was defined as following: high waist circumference (≥90 cm for men and ≥85 cm for women), high BP (systolic BP ≥130 or diastolic BP ≥80 mmHg or the use of antihypertensive medication), high glucose (≥100 mg/dL), high triglycerides (≥150 mg/dL or the use of a relevant medication), and low HDL-C (<40 mg/dL for men and <50 mg/dL for women or the use of a relevant medication).

© 2023 Park JC et al. *JAMA Network Open.*

eTable3. Association of cumulative income status and the number of income decreases with risk of incident type 2 diabetes further adjusted for lifestyle factors, obesity, and income in 2008.

	No. of participants	No. of events	Total no. of person-years of follow up	Incidence rate (per 1,000 person-years)	HR (95% CI)	
					Model 4[a]	Model 5[b]
Cumulative numbers of years of being in low-income quartile[c]						
0	5,218,630	230,469	32,498,913	7.1	1 (Ref.)	1 (Ref.)
1	835,652	37,955	5,194,246	7.3	1.07 (1.06, 1.08)	1.04 (1.03, 1.05)
2	546,814	25,274	3,398,630	7.4	1.08 (1.07, 1.10)	1.05 (1.03, 1.06)
3	400,853	19,443	2,487,821	7.8	1.11 (1.09, 1.13)	1.06 (1.05, 1.08)
4	317,724	16,351	1,968,269	8.3	1.12 (1.10, 1.14)	1.07 (1.05, 1.08)
5	501,554	30,439	3,086,354	9.9	1.18 (1.16, 1.19)	1.09 (1.08, 1.11)
P for trend					<.001	<.001
Cumulative numbers of years of being in very low-income status[c]						
0	7,684,335	348,915	47,803,647	7.3	1 (Ref.)	1 (Ref.)
1	20,840	1,537	127,291	12.1	1.37 (1.31, 1.45)	1.30 (1.24, 1.37)
2	18,835	1,474	114,696	12.9	1.37 (1.30, 1.44)	1.28 (1.21, 1.35)
3	15,090	1,145	91,943	12.5	1.41 (1.33, 1.49)	1.34 (1.26, 1.42)
4	15,285	1,148	93,003	12.3	1.40 (1.32, 1.48)	1.31 (1.24, 1.39)
5	66,842	5,712	403,653	14.2	1.44 (1.40, 1.48)	1.33 (1.29, 1.36)
P for trend					<.001	<.001
Cumulative numbers of years of being in high-income quartile[c]						
0	3,529,516	168,842	21,887,213	7.7	1 (Ref.)	1 (Ref.)
1	714,117	31,777	4,441,779	7.2	0.95 (0.94, 0.96)	0.98 (0.97, 0.99)
2	518,952	22,854	3,231,188	7.1	0.94 (0.92, 0.95)	0.97 (0.96, 0.99)
3	484,512	21,403	3,019,181	7.1	0.94 (0.92, 0.95)	0.98 (0.96, 0.99)
4	476,828	20,964	2,970,882	7.1	0.91 (0.90, 0.93)	0.96 (0.95, 0.98)
5	2,097,302	94,091	13,083,988	7.2	0.89 (0.88, 0.90)	0.96 (0.94, 0.97)
P for trend					<.001	<.001
Number of income decreases						
0	6,187,532	282,471	38,487,586	7.3	1 (Ref.)	1 (Ref.)
1	1,493,834	70,764	9,277,508	7.6	1.05 (1.04, 1.06)	1.05 (1.04, 1.06)
≥2	139,861	6,696	869,139	7.7	1.07 (1.04, 1.09)	1.09 (1.06, 1.12)
P for trend					<.001	<.001

Abbreviations: HR, hazard ratio; CI, confidence interval.
[a] Model 4: adjusted for age, sex, and residential location (urban, rural, or metropolitan), high glucose (≥100 mg/dL), high BP (systolic BP ≥130 or diastolic BP ≥80 mmHg or the use of antihypertensive medication), high triglycerides (≥150 mg/dL or the use of a relevant medication), and low HDL-C (<40 mg/dL for men and <50 mg/dL for women or the use of a relevant medication), additionally adjusted for smoking (never, former, or current), alcohol consumption (none, mild to moderate <30, or heavy ≥30 g/day), physical activity (none, non-regular, or regular), body mass index (<18.5, 18.5-<23, 23-<25, 25-<30, ≥30 kg/m^2), and high waist circumference (≥90 cm for men and ≥85 cm for women).
[b] Model 5: further adjusted for income in 2008.
[c] The number of times an individual was categorized for low-, very low-, or high-income status was counted every year from 2008 to 2012 (baseline year).

© 2023 Park JC et al. *JAMA Network Open.*

eTable4. Association of the number of income decreases with risk of incident type 2 diabetes further adjusted for the number of income increases

	No. of participants	No. of events	Total no. of person-years of follow up	Incidence rate (per 1,000 person-years)	HR (95% CI)
					Model 6[a]
Number of income decreases					
0	6,187,532	282,471	38,487,586	7.3	1 (Ref.)
1	1,493,834	70,764	9,277,508	7.6	1.05 (1.03, 1.05)
≥2	139,861	6,696	869,139	7.7	1.07 (1.04, 1.09)
P for trend					<.001

Abbreviations: HR, hazard ratio; CI, confidence interval.
[a] Model 6: adjusted for age, sex, and residential location (urban, rural, or metropolitan), high glucose (≥100 mg/dL), high BP (systolic BP ≥130 or diastolic BP ≥80 mmHg or the use of antihypertensive medication), high triglycerides (≥150 mg/dL or the use of a relevant medication), and low HDL-C (<40 mg/dL for men and <50 mg/dL for women or the use of a relevant medication), additionally adjusted for the number of income increases.

© 2023 Park JC et al. *JAMA Network Open.*

eTable 5. Association of cumulative income status and the number of income decreases with risk of incident type 2 diabetes, after excluding those with a prior history of cancer and cardiovascular disease

	No. of participants	No. of events	Total no. of person-years of follow up	Incidence rate (per 1,000 person-years)	Model 3[a]
					HR (95% CI)
Cumulative number of years of being in low-income quartile[b]					
0	5,077,382	220,670	31,638,753	7.0	1 (Ref.)
1	812,401	36,200	5,053,416	7.2	1.09 (1.08, 1.10)
2	531,029	24,061	3,303,173	7.3	1.10 (1.09, 1.12)
3	388,847	18,520	2,415,081	7.7	1.13 (1.11, 1.15)
4	307,992	15,534	1,909,648	8.1	1.14 (1.12, 1.16)
5	483,323	28,794	2,977,864	9.7	1.22 (1.20, 1.23)
P for trend					<.001
Cumulative number of years of being in very low-income status[b]					
0	7,473,807	333,767	46,524,032	7.2	1 (Ref.)
1	18,963	1,353	116,219	11.6	1.46 (1.38, 1.54)
2	17,509	1,339	106,860	12.5	1.49 (1.41, 1.57)
3	14,061	1,042	85,914	12.1	1.52 (1.43, 1.61)
4	14,231	1,026	86,826	11.8	1.48 (1.39, 1.57)
5	62,421	5,252	378,084	13.9	1.58 (1.54, 1.63)
P for trend					<.001
Cumulative number of years of being in high-income quartile[b]					
0	3,437,902	161,400	21,335,637	7.6	1 (Ref.)
1	694,946	30,334	4,325,494	7.0	0.95 (0.94, 0.96)
2	504,200	21,795	3,141,463	6.9	0.93 (0.92, 0.95)
3	470,532	20,433	2,934,093	7.0	0.93 (0.92, 0.94)
4	462,210	19,986	2,881,761	6.9	0.90 (0.89, 0.92)
5	2,031,184	89,831	12,679,487	7.1	0.86 (0.85, 0.86)
P for trend					<.001
Number of income decreases					
0	6,017,572	270,198	37,455,602	7.2	1 (Ref.)
1	1,448,067	67,242	9,000,574	7.5	1.06 (1.05, 1.07)
≥2	135,335	6,339	841,759	7.5	1.08 (1.05, 1.11)
P for trend					<.001

Abbreviations: HR, hazard ratio; CI, confidence interval.
[a] Model 3: adjusted for age, sex, and residential location (urban, rural, or metropolitan), high glucose (≥100 mg/dL), high BP (systolic BP ≥130 or diastolic BP ≥80 mmHg or the use of antihypertensive medication), high triglycerides (≥150 mg/dL or the use of a relevant medication), and low HDL-C (<40 mg/dL for men and <50 mg/dL for women or the use of a relevant medication).
[b] The number of times an individual was categorized for low-, very low-, or high-income status was counted every year from 2008 to 2012 (baseline year).

© 2023 Park JC et al. *JAMA Network Open.*

eTable 6. Five-year landmark analysis on the association of cumulative income status and the number of income decreases with risk of incident type 2 diabetes

	No. of participants	No. of events	Total no. of person-years of follow up	Incidence rate (per 1,000 person-years)	HR (95% CI)
					Model 3[a]
Cumulative number of years of being in low-income quartile[b]					
0	5,074,593	110,518	11,878,476	9.3	1 (Ref.)
1	810,908	17,965	1,895,801	9.5	1.08 (1.07, 1.10)
2	530,267	11,940	1,241,091	9.6	1.10 (1.08, 1.12)
3	388,001	9,180	907,447	10.1	1.13 (1.11, 1.16)
4	306,690	7,597	717,198	10.6	1.14 (1.11, 1.16)
5	479,309	13,678	1,120,734	12.2	1.19 (1.17, 1.21)
P for trend					<.001
Cumulative number of years of being in very low-income status[b]					
0	7,462,373	166,208	17,459,579	9.5	1 (Ref.)
1	19,639	697	46,240	15.1	1.44 (1.34, 1.55)
2	17,611	618	41,706	14.8	1.34 (1.24, 1.45)
3	14,144	473	33,348	14.2	1.36 (1.24, 1.49)
4	14,309	483	33,731	14.3	1.36 (1.25, 1.49)
5	61,692	2,399	146,143	16.4	1.45 (1.39, 1.50)
P for trend					<.001
Cumulative number of years of being in high-income quartile[b]					
0	3,416,888	79,609	7,970,336	10.0	1 (Ref.)
1	693,835	15,106	1,621,236	9.3	0.95 (0.94, 0.97)
2	504,493	10,891	1,180,808	9.2	0.94 (0.92, 0.96)
3	471,027	10,170	1,104,985	9.2	0.93 (0.91, 0.95)
4	463,624	9,982	1,087,078	9.2	0.91 (0.89, 0.93)
5	2,039,901	45,120	4,796,304	9.4	0.86 (0.85, 0.87)
P for trend					<.001
Number of income decreases					
0	6,006,823	134,382	14,058,130	9.6	1 (Ref.)
1	1,447,431	33,343	3,385,231	9.8	1.06 (1.05, 1.08)
≥2	135,514	3,153	317,387	9.9	1.09 (1.05, 1.13)
P for trend					<.001

Abbreviations: HR, hazard ratio; CI, confidence interval.
[a] Model 3: adjusted for age, sex, and residential location (urban, rural, or metropolitan), high glucose (≥100 mg/dL), high BP (systolic BP ≥130 or diastolic BP ≥80 mmHg or the use of antihypertensive medication), high triglycerides (≥150 mg/dL or the use of a relevant medication), and low HDL-C (<40 mg/dL for men and <50 mg/dL for women or the use of a relevant medication).
[b] The number of times an individual was categorized for low-, very low-, or high-income status was counted every year from 2008 to 2012 (baseline year).

© 2023 Park JC et al. *JAMA Network Open.*
