# Supplementary material for: Association of Sustained Low or High Income and Income Changes With Risk of Incident Type 2 Diabetes Among Individuals Aged 30 to 64 Years
Source: JAMA Netw Open. 2023 Aug 21;6(8):e2330024. doi: 10.1001/jamanetworkopen.2023.30024 (PMC10442710; doi:10.1001/jamanetworkopen.2023.30024)
Supplement: Supplement 2. — Data Sharing Statement [file jamanetwopen-e2330024-s002.pdf]

Data Sharing Statement

Park. Association of Sustained Low or High Income and Income Changes With Risk of Incident Type 2 Diabetes Among Individuals Aged 30 to 64 Years. *JAMA Netw Open*. Published August 21, 2023. doi:10.1001/jamanetworkopen.2023.30024

Data
Data available: No
